# An alternative resuscitation algorithm significantly reduces hands-of time during CPR: a full-scale simulation study

**DOI:** 10.1186/1757-7241-21-S2-A17

**Published:** 2013-09-09

**Authors:** Magnus Pedersen, Anna Mohammed, Lars Folkestad, Jacob Brodersen, Mikkel Brabrand

**Affiliations:** 1 Department of Medicine, Sydvestjysk Sygehus Esbjerg, Denmark; 2 Department of Endocrinology, Sydvestjysk Sygehus Esbjerg, Denmark; 3 Department of Gastroenterology, Sydvestjysk Sygehus Esbjerg, Denmark

## Background

A reduction in hands-off time during resuscitation leads to increased survival. We have previously shown that hands-off time can be reduced using our alternative cardio-pulmonary resuscitation (CPR) algorithm SOWS (Stop Only While Shocking), but only in small and limited simulations. We designed the present study to compare SOWS to the current European Resuscitation Council (ERC) 2010 guidelines in full-scale simulations. The aim was to decrease hands-off time.

## Methods

Using a randomized design, we compared SOWS to the 2010 ERC guidelines using predefined scenarios. In our algorithm, the defibrillator was charged while CPR was ongoing and compressions only interrupted for rhythm check. If a shock was required, it was delivered immediately and compressions resumed. A Laerdal Resusci® Anne and Lifepak 20 defibrillator were used. Hands-off time in percent of the entire cardiac arrest and compressions per minute were registered. Data will be presented as mean (standard deviation [SD]). Differences were tested using unpaired students t-test.

## Results

Thirty physicians participated (they had participated in 12-21 cardiac arrests and nine had completed an ALS course). We performed 11 full-scale simulations, six using 2010 ERC guidelines and five using SOWS. Mean hands-off time using ERC guidelines was 26.7 % (SD 4.3%) and 22.1 % (SD 2.3%) using SOWS, p = 0.02. Using ERC 2010 guidelines resulted in mean 83.8 (SD 13.7) compressions per minute and 95.0 (SD 2.4) compressions per minute with SOWS, p = 0.18.

## Conclusion

Using full-scale simulations, we demonstrated a significantly lower hands-off time when comparing SOWS to the 2010 ERC guidelines. Furthermore, an increase in compressions per minute where registered with our alternative algorithm, but this was not significant.

